# Primary breast lymphomas: a multicentric experience

**DOI:** 10.1186/1477-7819-8-53

**Published:** 2010-06-28

**Authors:** Nicola Avenia, Alessandro Sanguinetti, Roberto Cirocchi, Giovanni Bistoni, Stefano Trastulli, Fabio D'Ajello, Francesco Barberini, Giuseppe Cavallaro, Antonio Rulli, Angelo Sidoni, Giuseppe Noya, Giorgio De Toma, Francesco Sciannameo

**Affiliations:** 1 Endocrine Surgical Unit, University of Perugia, Perugia, Italy; 2 Department of General Surgery, University of Perugia, Perugia, Italy; 3 Plastic Surgery, University of Rome "La Sapienza", Rome, Italy; 4 Department of Surgery "P. Valdoni", University of Rome, Rome, Italy; 5 Institute of Pathological Anatomy, University of Perugia, Perugia, Italy

## Abstract

**Background:**

The Primary Breast Lymphomas (PBL) represent 0,38-0,70% of all non-Hodgkin lymphomas (NHL), 1,7-2,2% of all extranodal NHL and only 0,04-0,5% of all breast cancer. Most frequent PBLs are the diffuse large B cell lymphomas; in any case-reports MALT lymphomas lack or are a rare occurrence. Their incidence is growing. From 1880 (first breast resection for "lymphadenoid sarcoma" carried out by Gross) to the recent past the gold standard treatment for such diseases was surgery. At present such role has lost some of its importance, and it is matter of debate.

**Methods:**

Twenty-three women affected by PBL underwent surgery. Average age was 63 years (range: 39-83). Seven suffered of hypothyroidism secondary to autoimmune thyroiditis. Fourteen patients underwent mastectomy, nine patients received quadrantectomy (average neoplasm diameter: 1,85 cm, range: 1,1-2,6 cm). In 10 cases axillary dissection was carried out. Pathologic examination revealed 16 diffuse large B cell lymphomas and 7 MALT lymphomas.

**Results:**

Seven patients in the mastectomy group had a recurrence (50%), and all of them with diffuse large B cell lymphomas at stage II. Two of these had not received chemotherapy. No patient undergoing quadrantectomy had recurrence. In the mastectomy group disease free survival (DFS) at 5 and 10 years was 57 and 50%. Overall survival (OS) at 5 and 10 years was 71.4% and 57.1% respectively. All recurrences were systemic. DFS and OS at 5 and 10 years was 100% in the quadrantectomy group. In the patients with recurrence mortality was 85.7%. For stage IE DFS and OS at 5 and 10 years were 100%. For stage II DFS at 10 years was 62.5% and 56.2% respectively; OS at 5 and 10 years was 75% and 62.5% respectively. For MALT lymphomas DFS and OS at 5 and 10 years were 100%. For diffuse large B cell lymphomas DFS at 5 and 10 years was 62.5% and 56.2% respectively; OS at 5 and 10 years was 75% and 62,5% respectively.

**Conclusions:**

The role of surgery in this disease should be limited to get a definitive diagnosis while for the staging and the treatment CT scan and chemio/radioterapy are repectively mandatory. MALT PBLs have a definitely better prognosis compared to large B cell lymphomas. The surgical treatment must always be oncologically radical (R0); mastectomy must not be carried out as a rule, but only when tissue sparing procedures are not feasible. Axillary dissection must always be performed for staging purposes, so avoiding the risk of under-staging II o IE, due to the possibility of clinically silent axillary node involvement.

## Introduction

Non-Hodgkin lymphomas rarely affect the breast, the majority being primary (~60%) [1]. Up to now, 700 Primary Breast Lymphomas (PBL) have been described as case reports or case series [2-5]. PBLs generally affect women (only 10 cases have been reported in men) [6-9]. Their incidence is growing; recently Aviles and coll. presented a review consisting of 96 patients [10], while the International Extranodal Lymphoma Study Group (IELSG) has registered 204 cases [11].

In the past, different criteria have been used to define PBLs; presently the definition devised by Wiseman and Liao [12], and modified by Hugh and coll. is the one generally accepted in the Literature [13]:

• both mammary tissue and lymphomatous infiltrate present in close association in an adequate specimen;

• no evidence of widespread lymphoma by standard staging techniques or preceding extramammary lymphoma, although ipsilateral axillary node involvement is allowed if both lesions are present simultaneously.

From 1880 (first breast resection for "lymphadenoid sarcoma" carried out by Gross) to the recent past the gold standard treatment for such diseases was surgery [14]. At present such role has lost some of its importance, and it is matter of debate.

The endpoint of this study was to evaluate the role and the benefits (DFS and OS) of surgical treatment in the management of PLB.

## Methods

From January 1993 to December 2003 23 women affected by PBL underwent surgery. Average age was 63 years (range: 39-83). Seven suffered of hypothyroidism secondary to autoimmune thyroiditis. Sixteen cases presented with a palpable breast mass (11 on the right). Seven non-palpable lesions were diagnosed at routine mammography (5 on the right). In 1 case the mass infiltrated the skin. Axillary lymphoadenomegaly was present in a 83-year old patient. No patients complained of the typical lymphoma-associated symptoms: fever, night sweats, weight-loss. All patients were submitted to breast ultrasonography (US) and mammography; in 1 case a second nodule was found in a different quadrant of the same breast. FNAB, Core needle/Incision or excision biopsy of breast lump were performed for confirmation before proceeding. Bone marrow biopsy and total-body CT scanning were carried out on all patients. The diagnosis of PBL was preoperatively for all cases.

Fourteen patients underwent mastectomy (median neoplasm diameter: 5,5 cm, range: 3,5-8,7 cm) and 9 patients received quadrantectomy (median neoplasm diameter: 1,85 cm, range: 1,1-2,6 cm). The operative decisions (mastectomy vs quadrantectomy) were subordinated to diameter of neoplasms.

The axillary dissection was performed only in 10 patients underwent mastectomy; the exception ware patients presenting with clinically evident lymphoadenopathy and very elderly patients in not fit conditions.

Pathologic examination revealed 16 diffuse large B cell lymphomas and 7 MALT lymphomas. Eleven of the patients submitted to mastectomy had axillary node involvement by the disease (stage II according to Ann Arbor Classification).

No surgical complications were observed; in 6 case prolonged lymphatic drainage from the axillary dissection site occurred.

All patients - except 3 elderly subjects in poor general conditions (ASA III) - received systemic chemotherapy. In 2 cases radiotherapy was added.

## Results

Seven patients in the mastectomy group had a recurrence (50%), and all of them with diffuse large B cell lymphomas at stage II. Two of these had not received chemotherapy. No patient undergoing quadrantectomy had recurrence.

In the mastectomy group disease free survival (DFS) at 5 and 10 years was 57 and 50%. Overall survival (OS) at 5 and 10 years was 71.4% and 57.1% respectively. All recurrences were systemic. DFS and OS at 5 and 10 years was 100% in the quadrantectomy group. In the patients with recurrence mortality was 85.7%.

For stage IE DFS and OS at 5 and 10 years were 100%. For stage II DFS at 10 years was 62.5% and 56.2% respectively; OS at 5 and 10 years was 75% and 62.5% respectively. For MALT lymphomas DFS and OS at 5 and 10 years were 100%. For diffuse large B cell lymphomas DFS at 5 and 10 years was 62.5% and 56.2% respectively; OS at 5 and 10 years was 75% and 62,5% respectively.

## Discussion

PBLs represent 0,38-0,70% of all non-Hodgkin lymphomas (NHL), 1,7-2,2% of all extranodal NHL [15-19]; and only 0,04-0,5% of all breast cancer [16,18-23]. Most frequent PBLs are diffuse large B cell lymphomas (53%) [4]; in any case-reports MALT lymphomas lack or are a rare occurrence: 0 pts Bobrow [23], 0 pts Prévot [24], 4 pts (44%) Mattia [16], 7 pts (20%) Hugh [13], 0 pts Jeon [25], 2 pts (5%) Arber [19], and 9 pts (64%) Farinha [26]. Burkitt-type PBLs are a relatively rare entity, typical of young women.

Up to the recent past surgery was considered the gold standard in the management of breast lymphomas [22,27]. Presently such tenet has been challenged, and the role of surgery has lost part of its importance [20].

Scientific societies have proposed guide-lines in order to standardize therapeutic strategies. NCCN (National Comprehensive Cancer Network) offers different solutions based on histology and clinical stage according to the Ann Arbor Classification [28,29]. For MALT lymphomas in stage IE and II there isn't a treatment of choice. Surgery and Radiotherapy are equally effective in prognostic terms. Surgery - of course - affords a better histological evaluation. For all large B cell lymphomas the optimal treatment is chemotherapy. For MALT stage III and IV the preferred therapy is chemo-and radiotherapy. Furthermore, the ideal surgical technique is matter of debate. Some Authors carry out routinely mastectomy with axillary lymph-node dissection, others deem such intervention an overtreatment and perform tissue-sparing resections (quadrantectomy, tumorectomy), always associated with axillary lymph-node dissection and adjuvant chemo- radiotherapy. Finally, some do not believe in axillary dissection, so they don't perform it. In the review from Jeanneret - Sozzi adjuvant chemotherapy showed only scant advantages in terms of local control, and none in terms of OS and DFS [30].

Disagreement regarding the treatment of such disease stems from its rareness, with small case reports; consequently, randomized controlled trials or clinically controlled trials can not be carried out.

Jennings and coll. have recently published a review on 465 cases described in the Literature, and have showed that mastectomy does not afford better results concerning survival or recurrence [31]. More significant results were reported by Jeanneret-Sozzi and coll. in a multicentric study on 84 patients treated from 1970 to 2000; surgery is not a negative prognostic factor, on the contrary it turns out to be a positive prognostic index whenever a conservative procedure can be carried out [30]. Such evidence derives from the fact that - if technically feasible - a limited resection of the neoplasm is sufficient and that mastectomy is not able to change the outcome. Statistically significative negative prognostic factors are: tumor diameter (>4,5 cm) and lymphomatous dissemination to the axillary lymph-nodes [5].

## Conclusion

The role of surgery in this disease should be limited to get a definitive diagnosis while for the staging and the treatment CT scan and chemio/radioterapy are respectively mandatory. MALT PBLs have a definitely better prognosis compared to large B cell lymphomas. The surgical treatment must always be oncologically radical (R0); mastectomy must not be carried out as a rule, but only when tissue sparing procedures are not feasible. Axillary dissection must always be performed for staging purposes, so avoiding the risk of under-staging II o IE, due to the possibility of clinically silent axillary node involvement. Surgical excision enables adequate pathologic evaluation, not obtainable on FNA cytology, but provided by excisional biopsy. Furthermore, surgery affords excellent local tumor control, with clear advantages especially in elderly patients with large neoplasms involving the skin. Negative prognostic factors are: tumor diameter, lymphomatous dissemination to the axillary nodes.

The association between autoimmune thyroiditis and breast lymphomas - that we observed in seven cases, along with other Authors' reports - deserves to be investigated in the close future.

Nowadays, surgical resection plays a therapeutic role only in MALT lymphomas, whereas for large B cell lymphomas has only a diagnostic indication. For such disease, the cornerstone of treatment is systemic chemotherapy.

## Competing interests

The authors declare that they have no competing interests.

## Authors' contributions

All authors contributed equally to this work, read and approved the final manuscript.
